# Association of early neutrophil-to-lymphocyte ratio trajectories with cardiac recovery and live ICU discharge in patients with sepsis-induced cardiomyopathy: a retrospective cohort study

**DOI:** 10.3389/fmed.2026.1952142

**Published:** 2026-10-05

**Authors:** Baochao Pan, Ying Sun, Guiying Yang, Hepeng Fu, Xue Li, Yazhao Sun

**Affiliations:** 1 Department of Critical Care Medicine, Hebei Cangzhou Hospital of Integrated Traditional Chinese and Western Medicine, Cangzhou, Hebei, China; 2 Department of Hepatology, Cangzhou Third Hospital, Cangzhou, Hebei, China; 3 Department of Cardiology, Cangzhou People’s Hospital, Cangzhou, Hebei, China

**Keywords:** cardiac recovery, competing-risk analysis, live ICU discharge, neutrophil-to-lymphocyte ratio, sepsis-induced cardiomyopathy, trajectory analysis

## Abstract

**Background:**

Sepsis-induced cardiomyopathy (SIC) is characterized by potentially reversible myocardial dysfunction accompanied by a profound systemic inflammatory response. However, the prognostic significance of early dynamic changes in the neutrophil-to-lymphocyte ratio (NLR) in patients with SIC remains unclear. This study aimed to identify distinct early NLR trajectories and examine their associations with cardiac recovery and live discharge from the intensive care unit (ICU).

**Methods:**

This single-center retrospective cohort study included patients with SIC admitted to the Department of Critical Care Medicine, Hebei Cangzhou Hospital of Integrated Traditional Chinese and Western Medicine. NLR values were collected at ICU admission and at 24, 48, and 72 h thereafter. Latent class mixed modeling was used to identify distinct 72-h NLR trajectories. A 72-h landmark design was applied. The primary outcome was live ICU discharge within 28 days, with ICU death treated as a competing event and analyzed using Fine–Gray regression. The secondary outcome was cardiac recovery by days 5–7, defined as a follow-up left ventricular ejection fraction (LVEF) of ≥50% or an absolute increase of ≥10 percentage points from baseline. Multivariable logistic regression was used to evaluate the association between NLR trajectories and cardiac recovery.

**Results:**

A total of 510 patients contributed 2,040 longitudinal NLR measurements. Three distinct trajectories were identified: low-stable (*n* = 230, 45.1%), high-decreasing (*n* = 170, 33.3%), and persistent-high (*n* = 110, 21.6%). The geometric mean NLR changed from 7.85 to 6.16, from 18.47 to 8.01, and from 14.95 to 22.87, respectively, during the first 72 h. Live ICU discharge occurred in 85.7%, 67.1%, and 36.4% of patients in the three groups, respectively. Compared with the low-stable group, the adjusted subdistribution hazard ratios for live ICU discharge were 0.49 [95% confidence interval (CI), 0.38–0.63; *P* < 0.001] for the high-decreasing group and 0.25 (95% CI, 0.17–0.37; *P* < 0.001) for the persistent-high group. Cardiac recovery occurred in 80.4%, 61.8%, and 42.7% of patients, respectively. The corresponding adjusted odds ratios were 0.48 (95% CI, 0.29–0.79; *P* = 0.004) and 0.27 (95% CI, 0.14–0.51; *P* < 0.001). Associations with live ICU discharge were consistent across predefined subgroups. Septic shock showed an exploratory interaction with the association between NLR trajectories and cardiac recovery (P for interaction=0.034). Sensitivity analyses yielded similar results.

**Conclusions:**

Early 72-h NLR trajectories were independently associated with cardiac recovery and live ICU discharge in patients with SIC. A persistent-high trajectory identified patients with the poorest prognosis, whereas a high-decreasing trajectory was associated with intermediate outcomes. Serial assessment of NLR may provide a simple and clinically accessible tool for early risk stratification in SIC, although prospective external validation is warranted.

## Introduction

Sepsis is a life-threatening clinical syndrome caused by a dysregulated host response to infection and remains a leading cause of death worldwide (1, 2). Using data from the Global Burden of Disease Study, Rudd et al. estimated that approximately 48.9 million cases of sepsis occurred globally in 2017, resulting in 11.0 million deaths and accounting for 19.7% of all deaths worldwide (1). Myocardial dysfunction is a common manifestation of sepsis-related organ injury. In a serial assessment of 20 patients with septic shock, Parker et al. observed marked reductions in left ventricular ejection fraction (LVEF) and ventricular dilatation in some patients, with cardiac function recovering within approximately 10 days among survivors (3). In a systematic review and meta-analysis of 16 studies involving 2,132 patients, Hasegawa et al. reported a pooled prevalence of 20% for sepsis-induced cardiomyopathy (SIC). An analysis of 11 studies further showed that SIC was associated with an increased risk of short-term mortality (4). However, the condition may involve the left ventricle, right ventricle, or both ventricles, and the assessment of cardiac function may be influenced by volume status, vascular tone, mechanical ventilation, and vasoactive therapy. Consequently, its clinical phenotype and recovery course vary considerably between patients (5).

The pathophysiology of SIC involves multiple interacting mechanisms, including inflammatory mediator release, endothelial and microcirculatory dysfunction, abnormal nitric oxide production, impaired adrenergic responsiveness, disturbed calcium homeostasis, mitochondrial injury, and altered energy metabolism (6, 7). These changes occur within a host immune response that evolves rapidly over time. Sepsis may induce persistent neutrophil activation and delayed neutrophil apoptosis, together with lymphocyte apoptosis, lymphopenia, and impaired adaptive immunity (8). The neutrophil-to-lymphocyte ratio (NLR), derived from routine blood cell counts, provides an integrated measure of innate immune activation and relative lymphocyte depletion. It is inexpensive, readily available, and suitable for repeated assessment in critically ill patients (9). Because inflammatory and immune responses can change substantially during the early course of sepsis, a single NLR measurement may not distinguish transient elevation, treatment-related decline, and persistent elevation. Serial changes in NLR may therefore provide a more informative representation of disease evolution than an isolated value.

Previous studies have suggested that dynamic changes in NLR are associated with outcomes in sepsis. Riché et al. enrolled 130 patients with septic shock and monitored differential leukocyte counts during the first 5 days. They found distinct NLR patterns among patients who died early and those who died later (10). Lorente et al. included 203 patients with sepsis from three hospitals in Spain and measured NLR on days 1, 4, and 8 after diagnosis. Compared with 135 survivors, the 68 patients who died within 30 days had higher NLR values at each time point, and the association remained after accounting for concurrent Sequential Organ Failure Assessment (SOFA) score and lactate level (11). Liu et al. studied 216 patients with sepsis caused by intra-abdominal infection and serially assessed immune cell counts on days 1, 3, 5, and 7. NLR was consistently higher among the 72 non-survivors than among the 144 survivors (12). More recently, Arulkumaran et al. synthesized 30 studies involving 26,441 critically ill patients and found that higher NLR remained associated with mortality throughout the first week of critical illness (13). Nevertheless, previous evidence has largely been derived from general sepsis or septic shock populations, with mortality as the predominant outcome. Whether distinct early NLR patterns are related to cardiac recovery and the course of intensive care unit (ICU) treatment in patients with SIC remains unclear. Therefore, this study aimed to identify early 72-hour NLR trajectories in patients with SIC and to examine their associations with cardiac recovery and live ICU discharge within 28 days.

## Methods and materials

### Study design and population

This single-center retrospective cohort study included consecutive patients with SIC admitted to the Department of Critical Care Medicine, Hebei Cangzhou Hospital of Integrated Traditional Chinese and Western Medicine, between December 2023 and March 2026.

Patients were eligible if they were aged ≥18 years and had a diagnosis of SIC. Patients were excluded if they had pre-existing heart failure with reduced ejection fraction, severe valvular heart disease, or other structural heart disease that could substantially affect the assessment of cardiac function; acute myocardial infarction, acute myocarditis, stress cardiomyopathy, or a severe hematologic disorder; died or were discharged from the ICU before 72 h; or had insufficient longitudinal data for trajectory or outcome assessment, including missing complete blood count measurements at ICU admission or at 24, 48, or 72 h, missing echocardiography at baseline or on days 5–7, or missing prespecified covariate or outcome data. For patients with multiple eligible ICU admissions during the study period, only the first admission was retained for analysis. The patient selection process and reasons for exclusion are summarized in Figure 1.

**Figure 1:**
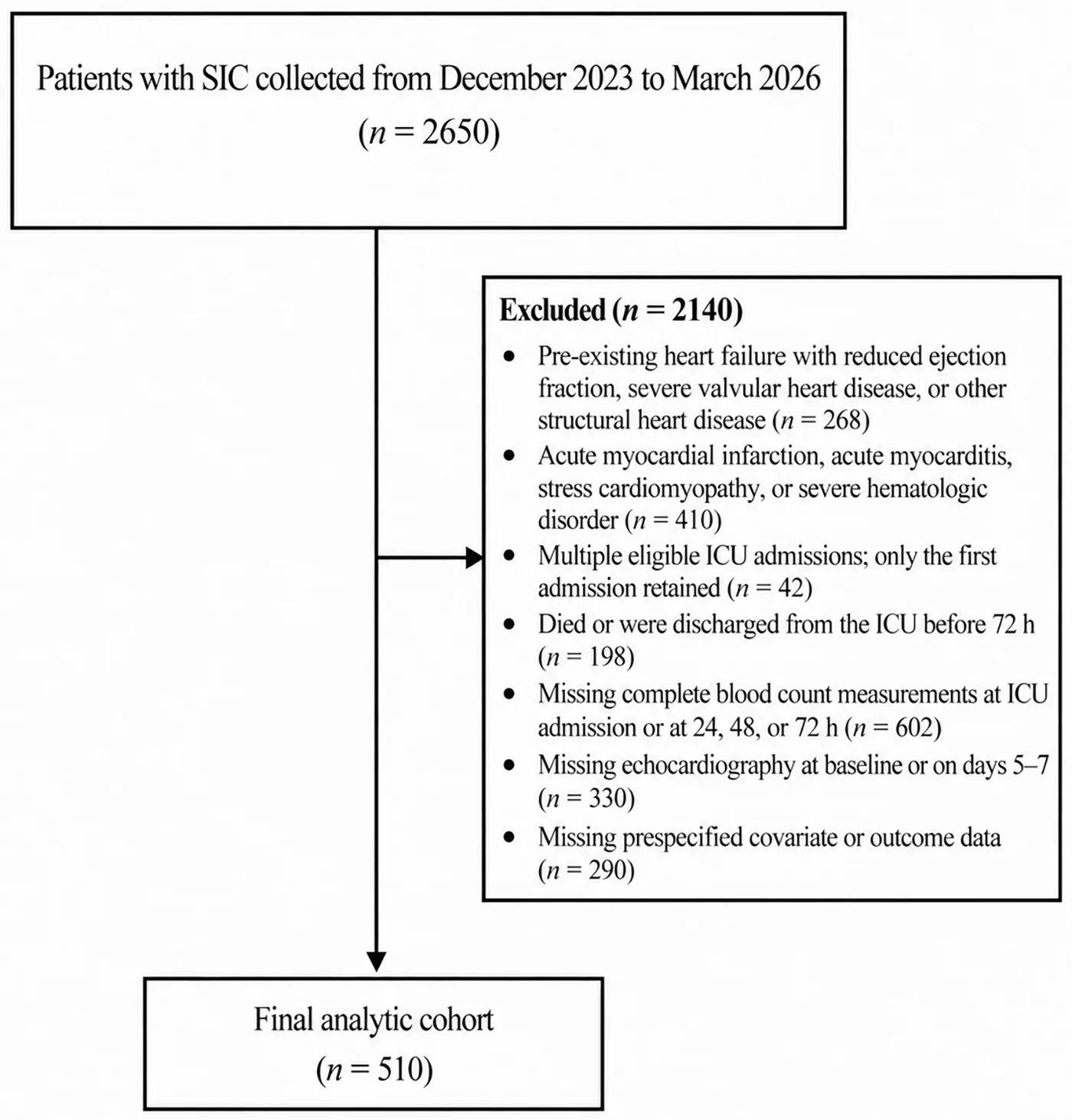
Flow chart of patient selection.

A 72-h landmark design was used because trajectory assignment required NLR measurements obtained during the first 72 h after ICU admission. Only patients who were alive and remained in the ICU at the landmark time were included, and follow-up for live ICU discharge began at that time. The study was approved by the Ethics Committee of Hebei Cangzhou Hospital of Integrated Traditional Chinese and Western Medicine (approval No. CZX2024058). The requirement for written informed consent was waived because of the retrospective study design. All data were de-identified before analysis.

### Data collection

Clinical data were extracted from the electronic medical record, ICU information system, laboratory information system, and echocardiography database. Demographic characteristics and comorbidities included age, sex, hypertension, coronary heart disease, diabetes mellitus, and chronic kidney disease. The primary site of infection was classified as pulmonary, abdominal, urinary, or other. Measures of illness severity and early treatment included septic shock, SOFA score, lactate concentration, serum creatinine, invasive mechanical ventilation, norepinephrine-equivalent dose, and corticosteroid use during the first 72 h. Doses of vasoactive agents were converted to norepinephrine-equivalent doses using standardized conversion methods.

Inflammatory and cardiac biomarkers included C-reactive protein (CRP), procalcitonin (PCT), high-sensitivity cardiac troponin I (hs-cTnI), and N-terminal pro-B-type natriuretic peptide (NT-proBNP). Echocardiographic variables included LVEF and tricuspid annular plane systolic excursion (TAPSE). Unless otherwise specified, baseline variables were defined using the first valid measurement obtained closest to ICU admission. These baseline measurements were used for covariate characterization in the primary analyses. Follow-up LVEF was incorporated into the definition of cardiac recovery.

### NLR measurement and trajectory modeling

NLR was calculated by dividing the absolute neutrophil count by the absolute lymphocyte count obtained from the same blood sample. NLR values were collected at ICU admission and at 24, 48, and 72 h. When more than one measurement was available around a scheduled time point, the value closest to that time point was used.

Because NLR was right-skewed, values were natural log-transformed before trajectory modeling. Latent class mixed models (LCMMs) were fitted to identify distinct longitudinal NLR patterns during the first 72 h. Log-transformed NLR was modeled as the dependent variable, with time entered as both a linear fixed effect and a class-specific effect. A patient-specific random intercept was included to account for within-patient correlation. Models containing one to four latent classes were fitted. For models with two or more classes, grid search with multiple random starting values was used to reduce the likelihood of convergence to a local maximum.

Model selection was based primarily on the Bayesian information criterion (BIC) and additionally considered the Akaike information criterion (AIC), entropy, class size, mean posterior probabilities, and clinical interpretability. Patients were assigned to the class with the highest posterior probability. Classification quality was assessed using the mean posterior probability within each class and the proportion of patients with a maximum posterior probability ≥0.70. For presentation, model estimates were exponentiated back to the original NLR scale and reported as geometric means with 95% confidence intervals (CIs).

### Outcomes

The primary outcome was live ICU discharge by day 28 after ICU admission. Live ICU discharge was defined as discharge from the ICU to a general ward or another non-ICU setting while the patient was alive. Follow-up began at the 72-h landmark and continued until live ICU discharge, death in the ICU, or day 28 after ICU admission, whichever occurred first. Live ICU discharge was treated as the event of interest, whereas ICU death was treated as a competing event. Patients who remained in the ICU at day 28 were administratively censored.

The secondary outcome was cardiac recovery on days 5–7 after ICU admission, defined as a follow-up LVEF ≥50% or an absolute increase in LVEF of at least 10 percentage points from baseline. When more than one echocardiographic examination was available during this interval, the examination closest to day 7 was used.

### Statistical analysis

Continuous variables were summarized as mean ± standard deviation or median with interquartile range, as appropriate, and compared using one-way analysis of variance or the Kruskal–Wallis test. Categorical variables were presented as counts and percentages and compared using the Pearson *χ*^2^ test or Fisher's exact test. The cumulative incidence of live ICU discharge was estimated for each NLR trajectory group and compared using Gray's test. Because ICU death precluded subsequent live ICU discharge, Fine–Gray subdistribution hazards models were used to estimate subdistribution hazard ratios (sHRs) and 95% CIs. The proportional subdistribution hazards assumption was assessed by including an interaction between trajectory group and the logarithm of follow-up time. When this interaction was significant, the sHR was interpreted as an average association over the follow-up period. The association between NLR trajectory and cardiac recovery was examined using binary logistic regression, with odds ratios (ORs) and 95% CIs reported. For both outcomes, unadjusted and multivariable-adjusted models were fitted, with the low-stable trajectory used as the reference group. Prespecified covariates were age, sex, hypertension, coronary heart disease, diabetes mellitus, chronic kidney disease, infection site, SOFA score, lactate, serum creatinine, invasive mechanical ventilation, norepinephrine-equivalent dose, and baseline LVEF. These covariates were selected to account for baseline clinical status and early illness severity. Serial changes in echocardiographic measures and other inflammatory or cardiac biomarkers were not included in the primary adjustment set, as the analysis focused on the association of NLR trajectories with subsequent outcomes beyond baseline status; follow-up LVEF was also incorporated directly into the definition of cardiac recovery.

Subgroup analyses were performed according to age (<65 vs. ≥65 years), sex, septic shock, invasive mechanical ventilation, SOFA score (<8 vs. ≥8), and baseline LVEF (<45% vs. ≥45%). The variable used to define each subgroup was omitted from the corresponding stratified model, while all other covariates were retained. Effect modification was assessed by adding an interaction term between trajectory group and the subgroup variable. These analyses were considered exploratory. Sensitivity analyses included a cause-specific Cox model for live ICU discharge, restriction of the analyses to patients with a maximum posterior probability ≥0.70, and use of a stricter definition of cardiac recovery requiring both a follow-up LVEF ≥50% and an absolute increase of at least 10 percentage points from baseline. Analyses were based on complete cases, consistent with the eligibility criteria requiring complete NLR measurements at the four prespecified time points, baseline and follow-up echocardiographic assessment, and complete key covariate and outcome data. All analyses were performed using R version 4.4.3 (R Foundation for Statistical Computing, Vienna, Austria). All tests were two-sided, and *P* < 0.05 was considered statistically significant.

## Results

### Study population and identification of early NLR trajectories

Among 2650 patients with SIC screened during the study period, 510 were included in the final analytic cohort, contributing 2040 longitudinal NLR measurements. LCMMs with one to four classes all converged. The three-class model had the lowest BIC (−1182.78), an entropy of 0.990, and no small class. Although the four-class model had a marginally lower AIC (−1227.30 vs. −1225.12), its BIC was higher (−1172.26), entropy was lower (0.945), and one class contained only 13 patients (2.5%). The three-class solution was therefore retained. The low-stable, high-decreasing, and persistent-high trajectories included 230 (45.1%), 170 (33.3%), and 110 (21.6%) patients, respectively. Average posterior probabilities were 0.994, 0.992, and 1.000, and 98.7%, 98.8%, and 100.0% of patients in the three groups had a maximum posterior probability of at least 0.70. Geometric mean NLR decreased from 7.85 to 6.16 in the low-stable group, from 18.47 to 8.01 in the high-decreasing group, and increased from 14.95 to 22.87 in the persistent-high group over the first 72 h (Figure 2).

**Figure 2:**
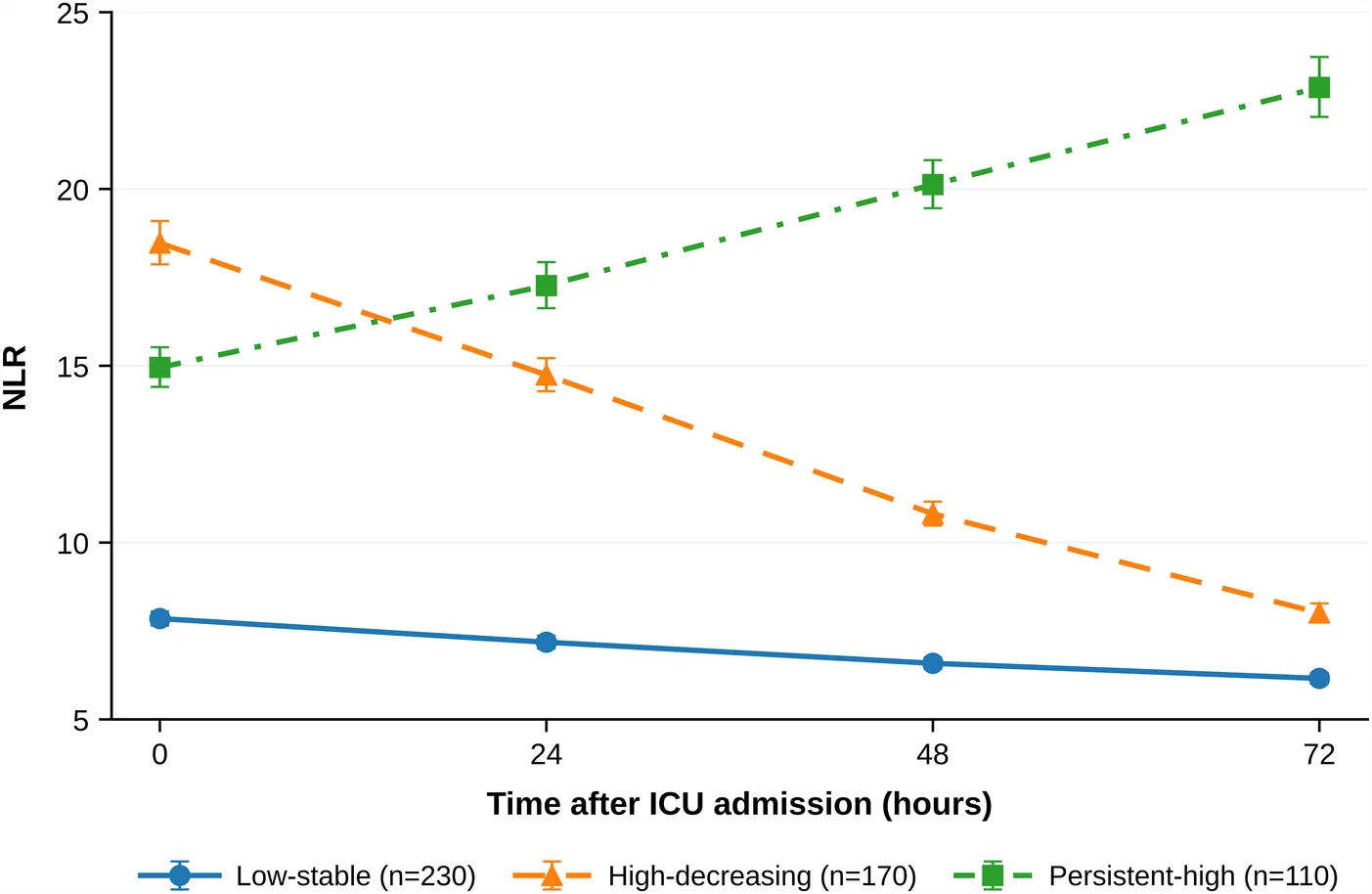
Early 72-hour NLR trajectories among patients with SIC.

Patients were assigned according to the maximum posterior probability from the three-class LCMM. Points and lines show geometric mean NLR values at 0, 24, 48, and 72 h; error bars show 95% CIs. *NLR*, neutrophil-to-lymphocyte ratio.

### Baseline characteristics according to NLR trajectory group

Baseline characteristics are summarized in Table 1. Age, sex, coronary heart disease, diabetes mellitus, and infection site were similar across trajectory groups. Hypertension and chronic kidney disease differed across groups (*P* = 0.032 and *P* = 0.004). Patients in the persistent-high group had the highest SOFA scores, lactate concentrations, serum creatinine levels, and norepinephrine-equivalent doses, together with the highest frequencies of septic shock and invasive mechanical ventilation (all *P* < 0.001). They also had the highest hs-cTnI and NT-proBNP concentrations and the lowest baseline LVEF and TAPSE values (all *P* < 0.001). Most measurements in the high-decreasing group were intermediate between the low-stable and persistent-high groups.

**Table 1:** Baseline characteristics and early treatment according to NLR trajectory group.

Variable	Overall (*n* = 510)	Low-stable (*n* = 230)	High-decreasing (*n* = 170)	Persistent-high (*n* = 110)	*P* value
**Demographic characteristics**					
Age, years	65.4 ± 12.2	64.4 ± 12.8	66.0 ± 11.8	66.7 ± 11.2	0.186
Male sex	294 (57.6%)	134 (58.3%)	98 (57.6%)	62 (56.4%)	0.947
**Pre-existing comorbidities**					
Hypertension	138 (27.1%)	50 (21.7%)	50 (29.4%)	38 (34.5%)	0.032
Coronary heart disease	102 (20.0%)	47 (20.4%)	36 (21.2%)	19 (17.3%)	0.710
Diabetes mellitus	140 (27.5%)	60 (26.1%)	41 (24.1%)	39 (35.5%)	0.095
Chronic kidney disease	66 (12.9%)	28 (12.2%)	14 (8.2%)	24 (21.8%)	0.004
**Infection characteristics and illness severity**					
Site of infection					0.320
Pulmonary	284 (55.7%)	124 (53.9%)	92 (54.1%)	68 (61.8%)	
Abdominal	101 (19.8%)	40 (17.4%)	40 (23.5%)	21 (19.1%)	
Urinary	73 (14.3%)	36 (15.7%)	24 (14.1%)	13 (11.8%)	
Other	52 (10.2%)	30 (13.0%)	14 (8.2%)	8 (7.3%)	
Septic shock	199 (39.0%)	55 (23.9%)	66 (38.8%)	78 (70.9%)	<0.001
SOFA score	8 (6, 9)	7 (5, 8)	8 (7, 10)	10 (8, 11)	<0.001
Lactate, mmol/L	2.55 (2.00, 3.04)	2.28 (1.73, 2.81)	2.54 (2.12, 2.97)	3.18 (2.66, 3.94)	<0.001
Serum creatinine, μmol/L	91.8 (71.1, 122.4)	87.1 (63.1, 110.3)	90.3 (73.2, 114.7)	111.0 (87.2, 166.1)	<0.001
**Organ support and early treatment**					
Invasive mechanical ventilation	260 (51.0%)	86 (37.4%)	96 (56.5%)	78 (70.9%)	<0.001
Norepinephrine-equivalent dose, μg/kg/min	0.00 (0.00, 0.11)	0.00 (0.00, 0.00)	0.00 (0.00, 0.11)	0.13 (0.00, 0.21)	<0.001
Corticosteroid use within 0–72 h	186 (36.5%)	68 (29.6%)	60 (35.3%)	58 (52.7%)	<0.001
**Inflammatory and cardiac biomarkers**					
Baseline CRP, mg/L	154.0 (114.1, 195.7)	111.9 (97.4, 132.5)	196.5 (172.1, 234.8)	181.0 (159.3, 209.1)	<0.001
Baseline PCT, ng/mL	5.65 (3.06, 8.30)	2.90 (2.32, 3.76)	8.35 (6.93, 10.43)	7.76 (6.29, 9.61)	<0.001
Baseline hs-cTnI, ng/L	93.0 (54.2, 155.1)	62.7 (42.1, 110.0)	103.5 (62.5, 145.2)	156.9 (96.1, 234.4)	<0.001
Baseline NT-proBNP, pg/mL	2,108.2 (1,202.2, 3,794.5)	1,345.1 (883.8, 2,446.1)	2,322.3 (1,359.1, 3,794.5)	3,959.2 (2,634.1, 6,683.6)	<0.001
**Echocardiographic parameters**					
Baseline LVEF, %	44.2 ± 3.0	45.4 ± 2.6	43.7 ± 2.8	42.3 ± 2.7	<0.001
Baseline TAPSE, mm	18.1 ± 1.9	18.9 ± 1.8	17.7 ± 1.7	17.0 ± 1.7	<0.001

CRP, C-reactive protein; hs-cTnI, high-sensitivity cardiac troponin I; LVEF, left ventricular ejection fraction; NT-proBNP, N-terminal pro-B-type natriuretic peptide; PCT, procalcitonin; SOFA, Sequential Organ Failure Assessment; TAPSE, tricuspid annular plane systolic excursion.

### Association between NLR trajectories and live ICU discharge within 28 days

After the 72-hour landmark, live ICU discharge by day 28 occurred in 197 of 230 patients (85.7%) in the low-stable group, 114 of 170 (67.1%) in the high-decreasing group, and 40 of 110 (36.4%) in the persistent-high group. The cumulative incidence functions differed across groups (Gray's test *P* < 0.001; Figure 3). Relative to the low-stable group, the unadjusted sHRs were 0.45 (95% CI, 0.35–0.56) for the high-decreasing group and 0.19 (95% CI, 0.14–0.27) for the persistent-high group. After multivariable adjustment, the corresponding sHRs were 0.49 (95% CI, 0.38–0.63; *P* < 0.001) and 0.25 (95% CI, 0.17–0.37; *P* < 0.001) (Table 2).

**Figure 3:**
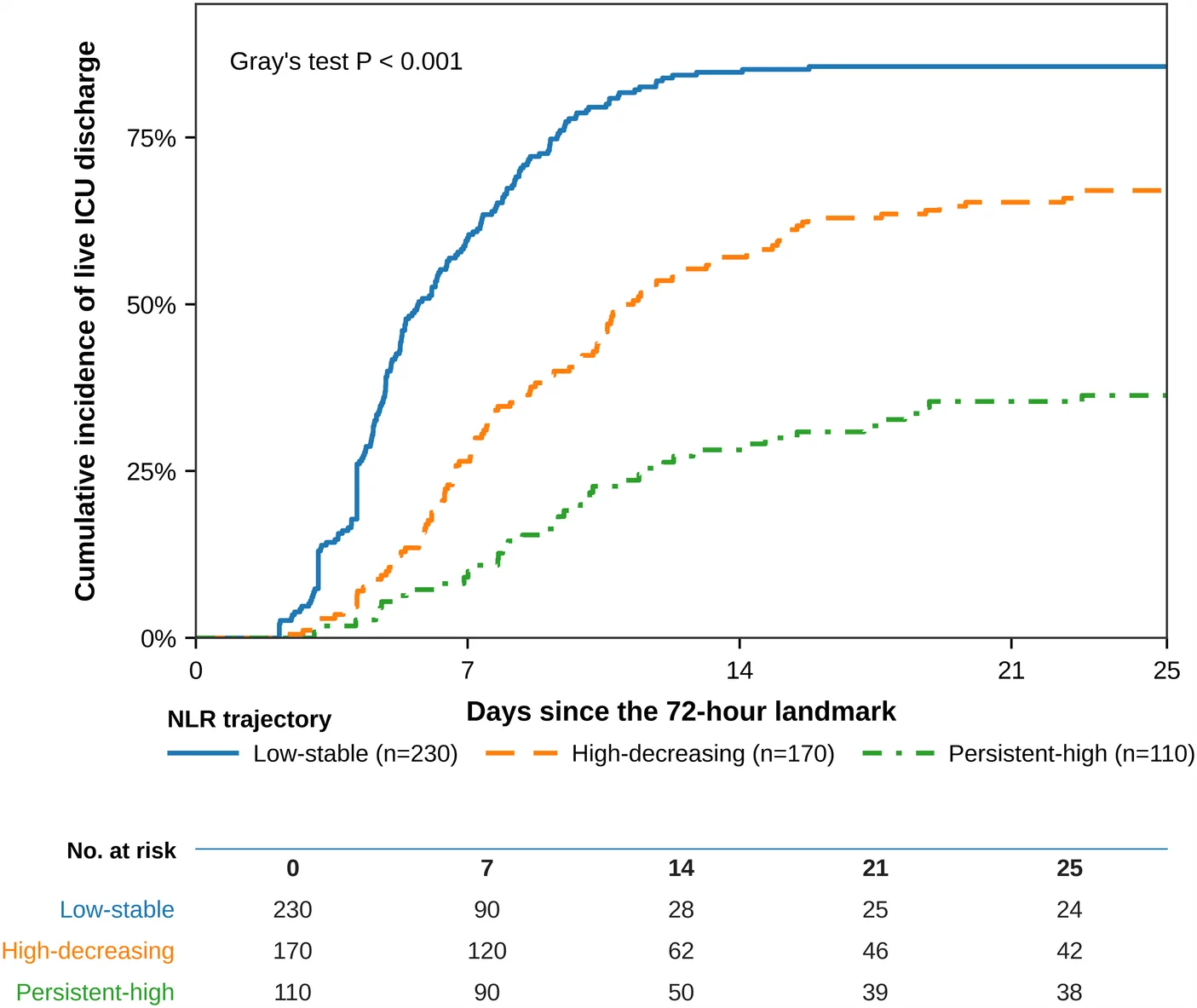
Cumulative incidence of live ICU discharge within 28 days according to NLR trajectory group.

**Table 2:** Association between NLR trajectories and live ICU discharge within 28 days.

NLR trajectory group	Live ICU discharge, n/N (%)	Unadjusted sHR (95% CI)	*P* value	Adjusted sHR (95% CI)	*P* value
Low-stable	197/230 (85.7%)	1.00 (Reference)		1.00 (Reference)	
High-decreasing	114/170 (67.1%)	0.45 (0.35–0.56)	<0.001	0.49 (0.38–0.63)	<0.001
Persistent-high	40/110 (36.4%)	0.19 (0.14–0.27)	<0.001	0.25 (0.17–0.37)	<0.001

sHRs were estimated using Fine–Gray models, with ICU death as a competing event and the low-stable group as the reference. The adjusted model included age, sex, hypertension, coronary heart disease, diabetes mellitus, chronic kidney disease, infection site, SOFA score, lactate, serum creatinine, invasive mechanical ventilation, norepinephrine-equivalent dose, and baseline LVEF. Because trajectory-by-log(time) interactions were significant, the sHRs represent average associations over follow-up.

### Association between NLR trajectories and cardiac recovery

Cardiac recovery by days 5–7 occurred in 185 patients (80.4%) in the low-stable group, 105 (61.8%) in the high-decreasing group, and 47 (42.7%) in the persistent-high group (overall *P* < 0.001). Using the low-stable group as the reference, the unadjusted ORs were 0.39 (95% CI, 0.25–0.62) and 0.18 (95% CI, 0.11–0.30), respectively. After multivariable adjustment, the ORs were 0.48 (95% CI, 0.29–0.79; *P* = 0.004) for the high-decreasing group and 0.27 (95% CI, 0.14–0.51; *P* < 0.001) for the persistent-high group (Table 3).

**Table 3:** Association between NLR trajectories and cardiac recovery.

NLR trajectory group	Cardiac recovery, *n* (%)	Unadjusted OR (95% CI)	*P* value	Adjusted OR (95% CI)	*P* value
Low-stable	185 (80.4)	1.00 (Reference)		1.00 (Reference)	
High-decreasing	105 (61.8)	0.39 (0.25–0.62)	<0.001	0.48 (0.29–0.79)	0.004
Persistent-high	47 (42.7)	0.18 (0.11–0.30)	<0.001	0.27 (0.14–0.51)	<0.001

Cardiac recovery was defined as follow-up LVEF ≥50% or an absolute increase in LVEF ≥10 percentage points by days 5–7. The low-stable group was the reference. The adjusted model used the same covariates as Table 2.

### Subgroup analyses

The associations with live ICU discharge were directionally consistent across subgroups defined by age, sex, septic shock, invasive mechanical ventilation, SOFA score, and baseline LVEF; no interaction test was significant (Figure 4A). For cardiac recovery, septic shock status modified the association (P for interaction=0.034), whereas the remaining interaction tests were not significant (Figure 4B). Among patients with septic shock, the adjusted ORs were 0.24 (95% CI, 0.09–0.63) for the high-decreasing group and 0.12 (95% CI, 0.04–0.33) for the persistent-high group. Among patients without septic shock, the corresponding estimates were 0.67 (95% CI, 0.35–1.26) and 0.55 (95% CI, 0.20–1.48). This interaction was considered exploratory.

**Figure 4:**
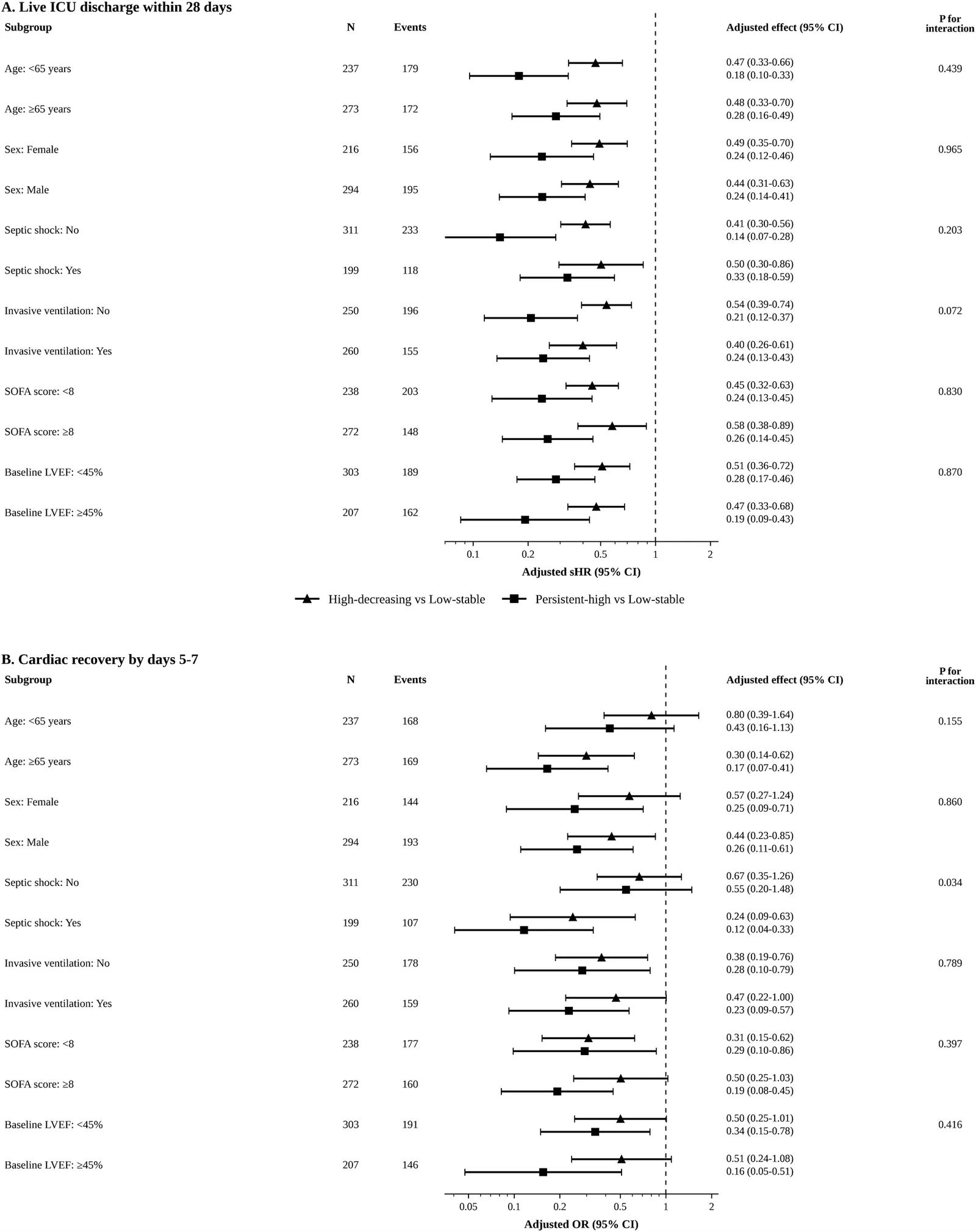
Subgroup analyses of the associations between NLR trajectories and clinical outcomes **(A)** shows live ICU discharge using fine–gray models; **(B)** shows cardiac recovery using logistic regression. The low-stable group was the reference. The adjusted model used the same covariates as Table 2. The variable defining each subgroup was omitted from the corresponding stratified model.

### Sensitivity analyses

In the cause-specific Cox model, the hazard ratios (HRs) for live ICU discharge were 0.49 (95% CI, 0.38–0.63) for the high-decreasing group and 0.29 (95% CI, 0.19–0.44) for the persistent-high group. When the Fine–Gray analysis was restricted to patients with a maximum posterior probability of at least 0.70, the sHRs were 0.49 (95% CI, 0.38–0.63) and 0.24 (95% CI, 0.16–0.37). For cardiac recovery, the corresponding ORs were 0.50 (95% CI, 0.30–0.82) and 0.27 (95% CI, 0.14–0.52) in the classification-certainty analysis, and 0.52 (95% CI, 0.33–0.82) and 0.30 (95% CI, 0.16–0.56) under the strict recovery definition.

## Discussion

In this cohort of 510 patients with SIC, three distinct NLR trajectories were identified during the first 72 h after ICU admission. Patients with a persistent-high trajectory had the lowest probability of cardiac recovery and the slowest live ICU discharge, whereas those with a high-decreasing trajectory had intermediate outcomes. After adjustment for demographic characteristics, comorbidities, illness severity, organ support, vasoactive therapy, and baseline LVEF, the effect estimates moved toward the null but remained statistically significant. This suggests that differences in baseline severity explained part, but not all, of the associations between NLR trajectories and clinical outcomes. The results were also consistent in the cause-specific analysis, the analysis restricted to patients with high classification certainty, and the analysis using a stricter definition of cardiac recovery. Taken together, these findings indicate that the direction and persistence of early NLR changes may provide clinically relevant information beyond a single NLR measurement.

The identified trajectories are biologically consistent with the evolving immune response during sepsis. Hyperinflammation and immune suppression can coexist, with persistent neutrophil activation and delayed neutrophil apoptosis occurring alongside lymphocyte apoptosis, depletion, and functional impairment (14). Drewry et al. reported that persistent lymphopenia on day 4 after sepsis diagnosis was independently associated with lower 28-day and 1-year survival, suggesting that failure of immune-cell recovery may be more informative than the initial lymphocyte count alone (15). Evidence regarding a single NLR measurement has been less consistent. Salciccioli et al. found that higher admission NLR was associated with mortality in an unselected critically ill population, although the association was not statistically significant in the sepsis subgroup (16). In contrast, a subsequent meta-analysis and sepsis-specific cohorts supported an association between elevated NLR and adverse outcomes (17, 18). Stoian et al. assessed NLR at ICU admission, 48 h, and 72 h in 120 patients and found that persistent elevation at 72 h was associated with increased mortality (19). Our findings extend this evidence by showing that patients with initially elevated NLR may follow markedly different courses. A progressive increase was associated with the least favorable outcomes, whereas a substantial decline was associated with partial, but not complete, risk reduction.

The association between NLR trajectory and cardiac recovery is particularly relevant in SIC. Patients in the persistent-high group had higher hs-cTnI and NT-proBNP concentrations, lower baseline LVEF and TAPSE, and greater circulatory and multiorgan dysfunction. The association with cardiac recovery nevertheless remained after adjustment for baseline LVEF and major indicators of illness severity. This may indicate that the NLR trajectory reflects an ongoing systemic process that is not fully captured by a single echocardiographic assessment. Pulido et al. evaluated 106 patients with severe sepsis or septic shock and found that myocardial dysfunction included overlapping patterns of left ventricular systolic dysfunction, left ventricular diastolic dysfunction, and right ventricular dysfunction (20). Landesberg et al. studied 262 patients and showed that diastolic dysfunction was associated with mortality and with elevated troponin and NT-proBNP concentrations (21). Orde et al. further demonstrated that myocardial strain imaging could identify ventricular impairment and prognostic differences that were not fully apparent from conventional measurements (22). A systematic review of speckle-tracking studies reached a similar conclusion, although substantial heterogeneity was present in the timing of echocardiography, strain thresholds, and patient selection (23). Right ventricular dysfunction also has prognostic importance and has been associated with both short-term and longer-term mortality in sepsis (24). Moreover, the classification of diastolic dysfunction varies according to the echocardiographic criteria applied, which complicates comparisons across studies (25). Lu et al. reported that different echocardiographic types of SIC were associated with distinct patterns of SOFA scores, cardiac biomarkers, and mortality (26). These findings support the interpretation that NLR trajectories may complement, rather than replace, repeated echocardiography and cardiac biomarker assessment.

The high-decreasing group warrants separate consideration. Although its geometric mean NLR declined from 18.47 at admission to 8.01 at 72 h, its rates of cardiac recovery and live ICU discharge remained lower than those of the low-stable group. A falling NLR may therefore indicate partial resolution of the inflammatory response without complete normalization of risk. The NLR at 72 h also remained higher than that in the low-stable group, and the consequences of the initial infectious burden, myocardial injury, and organ dysfunction may persist after the inflammatory marker begins to decline. By contrast, the rise from 14.95 to 22.87 in the persistent-high group may reflect failure of inflammatory resolution, continued physiological stress, inadequate infection control, or persistent lymphocyte depletion. The present data cannot distinguish among these mechanisms, and NLR should therefore be interpreted as a marker of the evolving host response rather than a direct mechanistic measure.

The trajectory-based findings are consistent with the broader recognition that sepsis cannot be adequately characterized by baseline measurements alone. Seymour et al. derived four clinical sepsis phenotypes with different patterns of inflammation, organ dysfunction, and mortality, demonstrating substantial heterogeneity among patients who fulfilled the same syndrome definition (27). Xu et al. identified and validated four 72-h SOFA trajectory subphenotypes, including rapidly worsening, delayed worsening, rapidly improving, and delayed improving patterns. Outcomes differed markedly even among patients with overlapping SOFA scores at ICU admission (28). Yang et al. similarly identified five longitudinal SOFA trajectories in 16,743 patients with sepsis and found substantial differences in mortality and other adverse outcomes across trajectory groups (29). Recent work involving 4,701 critically ill patients also showed that the depth and duration of early lymphopenia were associated with recurrent sepsis in a dose-dependent manner (30). These observations support the view that repeated measurements may distinguish patients whose initial values are similar but whose subsequent biological and clinical courses diverge. In the present study, live ICU discharge captured both survival and the pace of recovery from critical illness. The large separation between the cumulative incidence curves suggests that persistent NLR elevation was related not only to fatal events but also to delayed resolution of organ dysfunction and continued dependence on intensive care.

The associations with live ICU discharge were directionally consistent across the prespecified subgroups. Septic shock appeared to modify the relationship between NLR trajectory and cardiac recovery, with stronger associations observed among patients with shock. Severe vasoplegia, microcirculatory impairment, greater catecholamine exposure, and rapidly changing ventricular loading conditions may intensify the relationship between persistent immune dysregulation and myocardial dysfunction. This finding should, however, be interpreted cautiously because the analysis was exploratory, the confidence intervals were wider after stratification, and no correction was made for multiple interaction tests. From a clinical perspective, a persistent-high trajectory during the first 72 h may prompt closer reassessment of infection control, antimicrobial adequacy, hemodynamic status, organ support requirements, and cardiac function. NLR should not be used as a stand-alone treatment threshold because corticosteroids, catecholamines, renal dysfunction, hematologic conditions, secondary infection, and other physiological stressors can alter neutrophil and lymphocyte counts. Accordingly, NLR trajectories should be interpreted as markers of the evolving systemic host response rather than as cardiac-specific indicators of inflammation or myocardial injury.

Several limitations should be acknowledged. The retrospective, single-center design precludes causal inference and may limit generalizability. Requiring four NLR measurements and follow-up echocardiography may have introduced selection bias, particularly because patients with incomplete longitudinal measurements were excluded from the analytic cohort. Although NLR was assessed serially during the first 72 h, longitudinal changes in other inflammatory markers, cardiac biomarkers, and echocardiographic parameters such as TAPSE were not incorporated into the present analysis. These variables were primarily used to characterize baseline clinical severity and cardiac status, whereas NLR trajectory was the principal longitudinal exposure of interest. Follow-up LVEF was incorporated into the definition of cardiac recovery and was therefore not additionally modeled as a time-varying covariate. In addition, the 72-h landmark design excluded patients who died or left the ICU before the landmark, meaning that the findings apply primarily to patients who remained alive and in the ICU at 72 h. Residual confounding is also possible because the timing and appropriateness of antimicrobial therapy, adequacy of source control, cumulative fluid balance, transfusion, detailed corticosteroid exposure, and time-varying vasoactive support were not fully incorporated. NLR is a nonspecific composite marker, and the separate contributions of neutrophil expansion, lymphocyte depletion, redistribution, and treatment effects could not be determined. Cardiac recovery was primarily defined using LVEF, which is load dependent and does not fully characterize diastolic dysfunction, right ventricular dysfunction, or subclinical abnormalities detectable by myocardial strain. Prospective studies incorporating standardized serial assessment of inflammatory markers, cardiac biomarkers, and comprehensive echocardiographic parameters including myocardial strain imaging, together with molecular biomarkers, may help clarify whether their temporal changes provide additional information beyond NLR trajectories. Finally, latent trajectory classes are data driven. Their number and shape may vary according to patient characteristics, sampling schedules, laboratory practices, and model specification. Live ICU discharge may also be influenced by local bed availability and discharge policies. Because this cohort was derived from a single center and was restricted to patients who remained alive and in the ICU at the 72-h landmark, the generalizability of these trajectory patterns to other healthcare settings and to patients with very early death or ICU discharge remains uncertain. External validation in prospective multicenter cohorts is required before these trajectories can be used for routine clinical classification.

## Conclusion

Distinct NLR trajectories during the first 72 h after ICU admission were associated with cardiac recovery and live ICU discharge in patients with SIC. A persistent-high trajectory identified patients with the least favorable outcomes, whereas a high-decreasing trajectory was associated with an intermediate course. Serial NLR assessment may provide a readily available adjunct to clinical severity measures, cardiac biomarkers, and echocardiography. Prospective multicenter studies are needed to validate these trajectory patterns, clarify their biological basis, and determine whether they provide incremental value for clinical decision-making.

## Data Availability

The original contributions presented in the study are included in the article/Supplementary Material, further inquiries can be directed to the corresponding author.
